# A European Whitefish Linkage Map and Its Implications for Understanding Genome-Wide Synteny Between Salmonids Following Whole Genome Duplication

**DOI:** 10.1534/g3.118.200552

**Published:** 2018-10-08

**Authors:** Rishi De-Kayne, Philine G. D. Feulner

**Affiliations:** * Department of Fish Ecology and Evolution, Centre of Ecology, Evolution and Biogeochemistry, EAWAG Swiss Federal Institute of Aquatic Science and Technology, Switzerland; † Division of Aquatic Ecology and Evolution, Institute of Ecology and Evolution, University of Bern, Switzerland

**Keywords:** Coregonus, RAD, recombination rate, Salmonidae, sex-specific linkage maps, synteny

## Abstract

Genomic datasets continue to increase in number due to the ease of production for a wider selection of species including non-model organisms. For many of these species, especially those with large or polyploid genomes, highly contiguous and well-annotated genomes are still rare due to the complexity and cost involved in their assembly. As a result, a common starting point for genomic work in non-model species is the production of a linkage map. Dense linkage maps facilitate the analysis of genomic data in a variety of ways, from broad scale observations regarding genome structure *e.g.*, chromosome number and type or sex-related structural differences, to fine scale patterns *e.g.*, recombination rate variation and co-localization of differentiated regions. Here we present both sex-averaged and sex-specific linkage maps for *Coregonus sp. “Albock*”, a member of the European whitefish lineage (*C. lavaretus* spp. complex), containing 5395 single nucleotide polymorphism (SNP) loci across 40 linkage groups to facilitate future investigation into the genomic basis of whitefish adaptation and speciation. The map was produced using restriction-site associated digestion (RAD) sequencing data from two wild-caught parents and 156 F1 offspring. We discuss the differences between our sex-averaged and sex-specific maps and identify genome-wide synteny between *C. sp. “Albock*” and Atlantic Salmon (*Salmo salar*), which have diverged following the salmonid-specific whole genome duplication. Our analysis confirms that many patterns of synteny observed between Atlantic Salmon and *Oncorhynchus* and *Salvelinus* species are also shared by members of the Coregoninae subfamily. We also show that regions known for their species-specific rediploidization history can pose challenges for synteny identification since these regions have diverged independently in each salmonid species following the salmonid-specific whole genome duplication. The European whitefish map provided here will enable future studies to understand the distribution of loci of interest, *e.g.*, F_ST_ outliers, along the whitefish genome as well as assisting with the *de novo* assembly of a whitefish reference genome.

Although advances in sequencing technology continue to increase the yield and lower the cost of genomic data acquisition, the curation of this data into a usable format can still be challenging (Ellegren 2014). Understanding the relative positions of genetic markers is often essential for the detailed analysis of genomic datasets and is carried out in many model organisms by mapping reads to a reference genome (Sarropoulou 2011; Wolf and Ellegren 2017). However, marker ordering in the absence of a reference genome can also be carried out using a linkage map, which provides a measure of recombination distance rather than a physical distance, and as a result their production has become a common early step in the analysis of large genomic datasets (Lander and Green 1987; Lander and Schork 1994; Gross *et al.* 2008). Linkage maps are produced by observing recombination events which have occurred in parents by sequencing many offspring of that parental cross. Recombination events, which break up parental combinations of alleles, are used to assign markers to, and then order within, linkage groups, elucidating the relative location of thousands of markers along the genome (Sturtevant 1913; Rastas *et al.* 2013). The resulting maps hold information on the broad genome structure *e.g.*, number and length of linkage groups (*i.e.*, chromosomes) and can be used to evaluate synteny with related taxa to investigate genome evolution (Sarropoulou 2011; Hale *et al.* 2017; Leitwein *et al.* 2017). Linkage maps can be used to associate phenotypes and genotypes through quantitative trait locus (QTL) mapping (Doerge 2002). Linkage maps also hold the information to investigate the colocalization of regions under selection *e.g.*, F_ST_ outliers identified from genome scans and the recombination landscape itself (Sakamoto *et al.* 2000; Johnston *et al.* 2017). Empirical evidence has shown recombination to vary between species, populations, sexes and even individuals, highlighting the importance of its investigation in existing and new study organisms (Smukowski and Noor 2011; Kawakami *et al.* 2014; Stapley *et al.* 2017).

Linkage maps have become an essential tool in investigating evolution in non-model systems, providing information about the relative locations of markers along the genome and assisting in the assembly of new *de novo* genomes (Ellegren 2013; da Fonseca *et al.* 2016; Sutherland *et al.* 2016; Kubota *et al.* 2017; Sun *et al.* 2017; Zhigunov *et al.* 2017; Matz 2018). Many non-model organisms have specific ecological and evolutionary characteristics which make them particularly interesting for asking targeted evolutionary questions (Matz 2018). These features can include high speciation rate, remarkable numbers of species living in sympatry, high phenotypic and genomic diversity within or between populations, and unique ecological characteristics (Garvin *et al.* 2010; Ekblom and Galindo 2011; Hornett and Wheat 2012; Matz 2018). Carrying out studies to understand the genomic basis of these phenomena relies upon the development of new primary genomic resources in these non-model systems (Matz 2018). Linkage maps are therefore an ideal starting point to study evolution in new systems and open the door for the future production of more complex genomic resources including *de novo* genomes. Scaffolds produced during *de novo* genome assembly can be anchored to a linkage map, improving the contiguity and accuracy of the assembly (Fierst 2015; Lien *et al.* 2016; Feulner *et al.* 2018).

Salmonids are a particularly interesting family of teleost fishes in terms of their ecology and evolution, having colonized and adapted to a huge range of habitats, reflected in their diverse life history strategies (Nelson *et al.* 2006). They also have an interesting evolutionary history, influenced by a whole genome duplication which occurred 80-100 Mya in the shared ancestor of all salmonids (Macqueen and Johnston 2014; Lien *et al.* 2016). The family Salmonidae comprises of two main clades, which diverged ∼52 Mya (Macqueen and Johnston 2014). One clade is made up of the subfamily Salmoninae which includes salmon, trout and char species and the other contains the two subfamilies Thymallinae, containing grayling, and Coregoninae, containing whitefish and ciscos (Near *et al.* 2012; Macqueen and Johnston 2014). Following the salmonid-specific whole genome duplication the genome-wide pattern of rediploidization has varied across the genomes of different members of the Salmonidae family (Robertson *et al.* 2017). Many regions underwent cytological rediploidization in the ancestor of all salmonids and are referred to as ‘Ancestral Ohnologue Resolution’ (AORe) regions (Robertson *et al.* 2017). However, around a quarter of each salmonid genome rediploidized at a highly delayed rate, such that the major salmonid lineages (subfamilies) had been permanently separated by speciation before rediploidization was completed and those regions are known as ‘Lineage-specific Ohnologue Resolution’ (LORe) regions (Robertson *et al.* 2017). As ohnologue divergence depends on rediploidization, LORe regions have diverged into two duplicates independently in the different salmonid subfamilies, and consequently Atlantic Salmon and whitefish, for example, do not share direct orthology (Robertson *et al.* 2017).

Whitefish exhibit remarkable phenotypic diversity and high speciation rates, with multiple sympatric species having evolved post-glaciation in the last 15000 years (Lu and Bernatchez 1999; Kottelat and Freyhof 2007; Hudson *et al.* 2011). Two main whitefish species complexes exist, one in North America and the other in Europe. The North American whitefish complex comprises of *C. clupeaformis* species including sympatric ‘dwarf’ and ‘normal’ morphs which have arisen since the last glacial maximum (Bernatchez and Dodson 1990). The European species complex was previously described under the umbrella term ‘*C. lavaretus* species complex’, however ongoing work to formally describe the many species which are found across Europe is being undertaken by taxonomists (Douglas *et al.* 1999; Østbye *et al.* 2005; Kottelat and Freyhof 2007; Hudson *et al.* 2011). In Europe, whitefish are naturally found as far north as Finland and as far south as the Alps, with a particularly speciose monophyletic clade known as the Alpine whitefish which are distributed throughout Switzerland and its surrounding countries (Østbye *et al.* 2005; Hudson *et al.* 2011). Over 30 whitefish species have been described based on morphology in Switzerland alone (Steinmann 1950) and recent studies have identified additional cryptic diversity among sympatric whitefish, using genetic data to identify reproductively isolated species which have very similar morphology (Hudson *et al.* 2017; Doenz *et al.* 2018). Some lakes continue to harbor up to six sympatric whitefish species despite the reduction of genetic and phenotypic differences between many species and the extinction of others following lake eutrophication in the 1980s (Vonlanthen *et al.* 2012). Sympatric whitefish species are each-others closest relatives and thus monophyletic within unconnected Swiss lake systems and occupy a variety of ecological niches and exhibit a range of morphological differences (including body size, gill raker number and spawning season and depth; Douglas *et al.* 1999; Hudson *et al.* 2011; Vonlanthen *et al.* 2012; Hudson *et al.* 2017). It is the repeated ecological differentiation in sympatry that makes Swiss whitefish a particularly interesting radiation in which to study the genomic basis of adaptation. Although multiple studies have investigated the genetic basis of adaptation in other salmonids, those carried out on the European members of the Coregoninae subfamily are comparatively scarce.

The complex evolutionary history of salmonids, specifically the effect of the salmonid-specific whole genome duplication (Ss4R; Lien *et al.* 2016), makes the genetic basis of adaptation difficult to study in this family. Dense linkage maps have been produced to address these difficulties for a variety of Salmoninae, including Arctic Charr (Nugent *et al.* 2017), Brook Trout (Hale *et al.* 2017), Brown Trout (Leitwein *et al.* 2017) and Chinook Salmon (McKinney *et al.* 2016). These studies typically pair the use of dense linkage maps with the Atlantic Salmon (*Salmo salar*) reference genome to improve the genomic resolution of their analyses. However, due to the ∼50 million-year divergence time between Salmoninae and Coregoninae, and the limited number and density of whitefish linkage maps, the analysis of genomic whitefish datasets to answer questions about the physical distribution of loci and their function is limited (Rogers *et al.* 2001; Rogers and Bernatchez 2004; Rogers and Bernatchez 2007; Gagnaire *et al.* 2013). Only one whitefish linkage map produced using a restriction-site associated digestion (RAD) sequencing approach is available and was produced using data from North American whitefish (*C. clupeaformis*; Gagnaire *et al.* 2013). It includes 3438 single nucleotide polymorphism (SNP) markers resolved into 40 linkage groups (matching the karyotype of *C. clupeaformis*; Phillips and Rab 2007) and was successfully used to investigate expression QTL in *C. clupeaformis* (Gagnaire *et al.* 2013). However, studies which later described synteny patterns between salmonid genomes struggled to confidently resolve the relationships between lake whitefish linkage groups and other salmonid chromosomes using this map (Sutherland *et al.* 2016). The use of this map for investigating the remarkable European adaptive radiation of whitefish is further limited, due to the specificity of RAD markers and limited knowledge about genetic differentiation between *C. clupeaformis* and European whitefish species (*C. lavaretus* spp. complex) (Østbye *et al.* 2005; Hudson *et al.* 2011). The production of a European whitefish linkage map is therefore essential to study genome evolution within these extraordinary radiations.

In this study we produce a detailed linkage map for Alpine whitefish using a RAD sequencing approach. We produced both sex-specific and sex-averaged linkage maps for *Coregonus sp. “Albock*”, one member of the Alpine whitefish clade, from one F1 lab-bred cross. Here, we describe the sex-averaged and sex-specific linkage maps of *C. sp “Albock*” and use our sex-averaged linkage map to identify synteny between *C. sp. “Albock*” and Atlantic Salmon (*Salmo salar*). We identify rearrangements present between the two species which reflect the occurrence of fission and fusion events following the Ss4R whole genome duplication, some of which were confidently identified to be shared only between members of the Salmoninae subfamily in past studies. We also discuss the results of our synteny mapping in the context of the rediploidization history of salmonids. This *Coregonus* linkage map will facilitate future research regarding the genomic basis of adaptation in the adaptive radiation of Swiss whitefish and assist with the ongoing *de novo* assembly of the whitefish genome.

## Materials and Methods

### Experimental cross

One F1 family consisting of two parents and 156 offspring was used for linkage map construction. Both parent whitefish were sexually ripe, adult, *Coregonus sp. “Albock”*, a formally undescribed species which is one member of the European whitefish lineage (*C. lavaretus* spp. complex). *Coregonus sp. “Albock*” likely originates from an introduction of whitefish from Lake Constance into Lake Thun and taxonomic description of the species is in progress. The parental whitefish collected from Lake Thun in December 2016 were crossed *in vitro* by mixing sperm and eggs (obtained from the cantonal hatchery) together before adding cold water to harden successfully fertilized eggs. Fertilized eggs were then placed in a flow-through system which ran 5° lake water over the eggs for 11 weeks until they began to hatch. Before larvae had fully utilized their yolk sac they were sedated and killed with MS222 (50 mg/l for sedation; 200 mg/l for euthanization; buffered with sodium bicarbonate 500 mg/l) and preserved in 100% ethanol (February 2017; Animal Permit number LU03/15).

### DNA extraction, library preparation and sequencing

DNA for both parental whitefish was extracted from muscle tissue. Progeny DNA was extracted following the digestion of 176 whole larvae. Both parent and progeny DNA was extracted using DNeasy Blood and Tissue extraction kit (Qiagen). The DNA concentration of each extract was measured using the Qubit 1.0 Fluorometer (Thermo Fisher). In total five RAD libraries were made, with 44 F1 samples pooled into each of the four offspring RAD libraries and the two parental samples pooled into a fifth library. Each library was produced following the protocol of Baird *et al.* (2008) with slight modifications. The DNA concentration of each individual was normalized prior to the restriction enzyme digestion step to ensure 1 µg DNA was included for each F1. Since the parental library contained only two individuals, to achieve higher sequencing depth, 18 µg DNA from each parent was used for the digestion. Pre-digestion DNA integrity and the success of enzyme digestion was confirmed by running a subset of samples on a 1.4% agarose gel before and after enzyme digestion. The restriction enzyme digestion was carried out using the *Sbf-1* enzyme, which has been shown to digest salmonid DNA effectively (Gonen *et al.* 2014; Gagnaire *et al.* 2013; Sutherland *et al.* 2016), before the digested genomic DNA was ligated to individual-specific barcodes and the forward Illumina adaptor. Size selection after shearing took place using a SageELF to retain only DNA fragments between 300 and 700 base pairs (bp). Fragments were then amplified in a PCR after the ligation of the reverse Illumina adaptor. Each library was spiked with PhiX DNA (∼10% of reads) before being single-end sequenced, each on a single lane of Illumina HiSeq 2500 with 100 cycles at the Lausanne Genomic Technologies Facility (Switzerland).

### Sequence processing and genotyping

The first step of processing the 100 bp sequenced reads was to remove all PhiX reads using a Bowtie2 mapping approach (using default parameters except for the number of allowed mismatches which we set to 1; Langmead and Salzberg 2012). Next, all reads from the parental library were filtered for quality using Trimmomatic v.0.35 (Bolger *et al.* 2014). Bases were trimmed from the beginning and end of reads if they were below quality 3, a sliding-window approach was used with a 4 base wide window to trim bases below a quality score of 15. Reads were only retained if they had an average quality of 30 and if they were longer than 50 bp. Reads from the parental library and four offspring libraries were then demultiplexed and offspring reads were trimmed to 90 bp using the *process_radtags* module in Stacks version 1.40 (Catchen *et al.* 2013). Next, 20 offspring with < 1 million reads were discarded to leave both parents and 156 F1 offspring for analysis. A *de novo* reference assembly was produced by combining only reads from both parents, running the *ustacks* module in Stacks (Catchen *et al.* 2013) to identify putative SNP loci present in the parents of the cross (with a minimum coverage depth of 20) and the concatenation of these consensus stacks (Catchen *et al.* 2013). An index of this reference was then produced with Bowtie2 (Langmead and Salzberg 2012). Both parental and all offspring FASTA files were aligned to the parental *de novo* reference assembly using Bowtie2 (using default parameters except for the number of allowed mismatches which we set to 1) resulting in individual alignment files. The GATK *Haplotype Caller* (Poplin *et al.* 2017) was used to call genotypes, producing a VCF file retaining only SNPs genotyped with a minimum base quality score of 20 and a minimum confidence threshold of 20, *i.e.*, p-error 0.01. The use of GATK allowed us to further filter this genotype file with VCFtools (Danecek *et al.* 2011) to leave 20635 biallelic SNPs with a minimum phred quality score of 30 with indels removed. Since only one generation of offspring are included in an F1 linkage map, the most informative loci are those that are heterozygous in one parent and homozygous in the other (*e.g.*, maternal Aa, paternal aa or maternal aa, paternal Aa). Offspring can therefore be heterozygous or homozygous (*e.g.*, Aa or aa in an expected ratio of 1:1) and the phasing/origin of each allele is known. In addition to these highly informative loci, loci for which both parents are heterozygous can also provide information in the offspring in certain linkage mapping programs (*e.g.*, maternal Aa, paternal Aa). In these cases, three offspring genotypes may be observed *e.g.*, AA, Aa, aa in an expected ratio of 1:2:1 with only homozygous offspring being informative since we know that one copy of each allele is from each parent (*e.g.*, AA offspring or aa offspring have received one A from each parent or one a from each parent, respectively). Heterozygous offspring genotypes are uninformative since the origin of each allele is unknown (*e.g.*, Aa offspring may have received A or a from either parent). Loci were then filtered in R (R Core Team 2014) leaving only informative loci segregating in these two ways as well as removing any loci with missing data in either parent. All SNPs from RAD loci with more than three SNPs were removed and one SNP was chosen at random from those RAD loci with two SNPs. Remaining loci with over 20% missing data were also removed using R (R Core Team 2014), leaving 9757 loci for linkage mapping.

### Linkage mapping

Linkage map construction was carried out using Lep-MAP3 (Rastas 2017). First custom R and python scripts were used to convert the VCF file containing informative loci to Lep-MAP3 format before it was converted to a genotype likelihood table using the script linkage2post.awk and the *Transpose* module (Lep-MAP2; Rastas *et al.* 2015). Next Lep-MAP3 modules were used starting with the *ParentCall2* module identifying 7800 informative markers. The *Filtering2* module was then used to remove markers with significant segregation distortion (dataTolerance = 0.001). Linkage groups were then identified using *SeparateChromosomes2* with a logarithm of odds (LOD) score of 16 (lodLimit = 16) and the minimum number of markers per linkage group set to 25, resolving 40 linkage groups (corresponding to the 40 whitefish chromosomes identified by karyotyping; Phillips and Rab 2007) containing 5395 loci before within-group ordering of markers was carried out (Rastas 2017). Due to the slight stochastic variation in marker distances between runs, the *OrderMarkers2* module was used, specifying a sex-specific map (sexAveraged = 0), three times on each linkage group to produce a male and a female linkage map. This procedure was then repeated specifying a sex-averaged map (sexAveraged = 1). The marker orders with the highest likelihoods for each linkage group for each type of map were combined to produce the final most likely male and female sex-specific maps and one final sex-averaged map, each positioning the same 5395 SNP markers. A custom R script was used to calculate differences in the marker densities and lengths between maps and the sex-averaged map was plotted using MapChart (Voorrips 2002; R Core Team 2014).

### Synteny analysis

To identify synteny between the 29 Atlantic Salmon chromosomes and the 40 whitefish linkage groups, the *de novo* assembled RAD loci which were produced using the reads of the two parents of the cross, were mapped to the *Salmo salar* genome using Stampy v. 1.0.22 (Lunter and Goodson 2011) to produce an alignment file for all reference loci. Since whitefish and Atlantic Salmon are ∼52 million years divergent and transcript analysis has shown them be 93% similar, a divergence percentage of 7% (substitution rate = 0.07) was specified during mapping (Koop *et al.* 2008). A custom R script was then used to match the 5395 RAD loci within the complete sex-averaged map to the corresponding loci in the reference whitefish - Atlantic Salmon alignment file, extracting the salmon chromosome, base pair position and mapping quality. Mapped loci were then stringently filtered by their mapping quality score (MAPQ > 30) and the salmon chromosome with the most hits was noted. Linkage groups were then ordered to reflect their synteny with salmon chromosomes (Table 1) and renamed with the prefix ‘W’ to match salmon chromosome ordering. Synteny was visualized using the *circlize* package (Gu *et al.* 2014) in R plotting all links from reads with MAPQ > 30 to the corresponding salmon chromosome arm and position within each chromosome arm (Figure 2). To investigate the distribution of mappings within the salmon genome, specifically why some chromosome arms had few mappings, the rediploidization history of those arms was taken into account. Chromosome arms were classified as either AORe (n = 30) or LORe (n = 14) based on when in the salmonid lineage rediploidization occurred (from Robertson *et al.* 2017). Chromosome arms which had some minor proportion of LORe within a largely AORe chromosome arm (Ssa3p, Ssa5p, Ssa9qb, Ssa13qa, Ssa15qb and Ssa23) were excluded. An expected number of mappings was calculated for each chromosome arm based on the arm length relative to the sum of all arm lengths and the total number of mappings included in our synteny map. A ratio of expected/observed mappings was then calculated for each chromosome arm and plotted (with the exception of Ssa8q because of its infinite value resulting from 0 observed mappings), grouping chromosome arms by their mode of rediploidization (Figure 3). A Wilcoxon rank sum test was carried out to test whether expected/observed mapping ratios for AORe and LORe chromosome arms were significantly different.

**Table 1:** Table comparing statistics for the sex-averaged, female and male *C. sp. “Albock*” linkage maps. The results of synteny analysis are included, showing the homologous Atlantic Salmon chromosome (Ssa) for each whitefish linkage group (Calb) and the re-ordered whitefish linkage group name (W)

Whitefish Linkage Group	Number of SNPs	LG length (cM)	SNPs/cM	Female LG length (cM)	Female SNPs/cM	Male LG length (cM)	Male SNPs/ cM	Homologous Salmon Chromosome	Reordered Whitefish LG	Female:Male recombination ratio
Calb01	253	75.96	0.30	91.07	0.36	63.67	0.25	Ssa01	W02	1.43
Calb02	228	83.57	0.37	101.33	0.44	69.58	0.31	Ssa01	W03	1.46
Calb03	220	78.51	0.36	84.40	0.38	87.95	0.40	Ssa21	W32	0.96
Calb04	214	58.45	0.27	66.69	0.31	50.05	0.23	Ssa10	W15	1.33
Calb05	190	66.93	0.35	63.63	0.33	71.66	0.38	Ssa12	W18	0.89
Calb06	187	53.16	0.28	70.69	0.38	37.88	0.20	Ssa13	W20	1.87
Calb07	181	71.53	0.40	68.13	0.38	88.06	0.49	Ssa04	W06	0.77
Calb08	173	52.28	0.30	56.37	0.33	45.30	0.26	Ssa10	W14	1.24
Calb09	170	79.41	0.47	73.03	0.43	91.75	0.54	Ssa07	W10	0.80
Calb10	165	62.43	0.38	60.45	0.37	65.05	0.39	Ssa01	W01	0.93
Calb11	164	65.01	0.40	64.04	0.39	66.05	0.40	Ssa11	W16	0.97
Calb12	164	51.09	0.31	70.15	0.43	30.22	0.18	Ssa22	W33	2.32
Calb13	162	69.34	0.43	71.26	0.44	63.49	0.39	Ssa29	W40	1.12
Calb14	157	65.11	0.41	61.78	0.39	72.14	0.46	Ssa13	W19	0.86
Calb15	156	64.90	0.42	63.19	0.41	71.73	0.46	Ssa16	W24	0.88
Calb16	154	56.17	0.36	55.30	0.36	65.75	0.43	Ssa20	W31	0.84
Calb17	151	65.53	0.43	69.40	0.46	61.63	0.41	Ssa23	W34	1.13
Calb18	149	61.50	0.41	65.22	0.44	62.38	0.42	Ssa09	W11	1.05
Calb19	147	62.15	0.42	68.25	0.46	55.50	0.38	Ssa14	W21	1.23
Calb20	144	66.36	0.46	79.08	0.55	56.52	0.39	Ssa27	W37	1.40
Calb21	143	71.78	0.50	69.37	0.49	83.01	0.58	Ssa25	W36	0.84
Calb22	137	71.12	0.52	74.56	0.54	67.96	0.50	Ssa03	W04	1.10
Calb23	127	64.80	0.51	68.96	0.54	69.78	0.55	Ssa06	W09	0.99
Calb24	127	52.57	0.41	58.54	0.46	54.23	0.43	Ssa15	W22	1.08
Calb25	124	57.74	0.47	61.62	0.50	60.81	0.49	Ssa24	W35	1.01
Calb26	123	64.59	0.53	70.67	0.57	62.12	0.51	Ssa19	W29	1.14
Calb27	118	46.03	0.39	61.06	0.52	30.24	0.26	Ssa18	W27	2.02
Calb28	115	59.05	0.51	63.68	0.55	59.73	0.52	Ssa15	W23	1.07
Calb29	114	62.40	0.55	61.31	0.54	70.58	0.62	Ssa09	W12	0.87
Calb30	112	62.75	0.56	68.12	0.61	63.96	0.57	Ssa05	W08	1.07
Calb31	111	53.35	0.48	63.62	0.57	42.48	0.38	Ssa20	W30	1.50
Calb32	104	56.67	0.54	63.47	0.61	53.94	0.52	Ssa18	W28	1.18
Calb33	97	67.73	0.70	70.46	0.73	66.40	0.68	Ssa09	W13	1.06
Calb34	79	61.12	0.77	71.34	0.90	62.97	0.80	Ssa03	W05	1.13
Calb35	56	36.88	0.66	55.57	0.99	21.14	0.38	Ssa28	W38	2.63
Calb36	45	24.18	0.54	15.92	0.35	30.75	0.68	Ssa17	W26	0.52
Calb37	37	27.48	0.74	34.82	0.94	21.51	0.58	Ssa11	W17	1.62
Calb38	34	11.86	0.35	0.00	0.00	24.01	0.71	Ssa16	W25	0.00
Calb39	32	17.17	0.54	0.00	0.00	33.66	1.05	Ssa04	W07	0.00
Calb40	31	15.20	0.49	23.55	0.76	7.41	0.24	Ssa28	W39	3.18
Total	5395	2293.86		2460.10		2263.05				
Average	134.88	57.35	0.46	61.50	0.48	56.58	0.46			1.09

### Data availability

Fastq files for all 156 offspring and both parents are deposited in the NCBI short read archive (SRA accession PRJNA478121). All R, Python and bash scripts used can be accessed at https://github.com/RishiDeKayne/. Supplemental material including the genotype file (VCF), the Lep-MAP inpute file and all three linkage maps are available at Figshare: https://doi.org/10.25387/g3.7093799.

## Results and Discussion

### Linkage mapping

**Figure 1:**
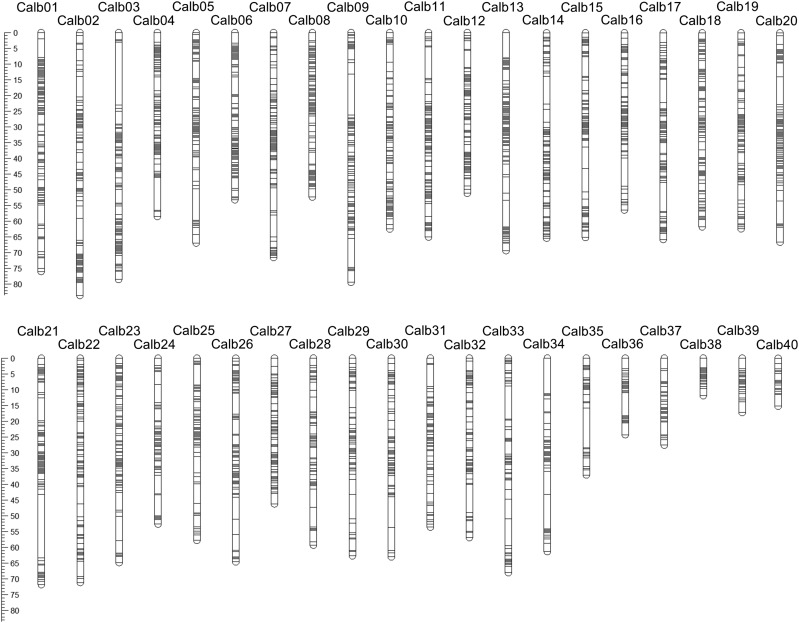
*Coregonus sp. “Albock*” (European whitefish species complex) linkage map showing the grouping and position of 5395 SNPs within a sex-averaged linkage map. The length of each of the 40 linkage groups is indicated by the scale in cM with linkage groups ordered by marker number from highest to lowest.

Our F1 cross was produced by crossing two wild *C. sp. “Albock*” adults. Both parents and 156 F1 offspring were successfully genotyped using a RAD-seq approach. In total 9757 SNPs were retained following stringent quality control and loci filtering steps, with 7800 identified as informative in Lep-MAP3 (Rastas 2017). Finally, 5395 SNPs were assigned to, and arranged within, linkage groups in both sex-averaged and sex-specific maps (Table 1; Figure 1). With the LOD score of 16, 40 linkage groups, corresponding to the 40 chromosomes observed in karyotype studies of the closely related European whitefish (*C. lavaretus*; Phillips and Rab 2007), were formed with an average of 135 markers per linkage group (Table 1). Map lengths varied from 2293.86 cM in the sex-averaged map to 2460.10 cM and 2263.05 cM in the female and male maps, respectively. All three maps produced in this study were considerably shorter than a previously published *C. clupeaformis* linkage map containing 3438 RAD markers, which had a total map length of 3061 cM (Gagnaire *et al.* 2013). Our sex-averaged *C. sp. “Albock*” map had an average linkage group length of 57.35 cM with the female and male sex-specific maps showing average linkage group lengths of 61.50 cM and 56.58 cM, respectively.

The number of SNPs per linkage group varied from 31 to 253 and the lengths of linkage groups varied from 15.20 cM to 83.57 cM in the sex-averaged map. Two linkage groups, Calb38 and Calb39, were comprised only of male-informative loci and therefore had lengths of 0 cM in the female map, with the longest linkage group in the female map being Calb02 at 101.33 cM. In the male map linkage groups vary in length from 7.41 cM to 88.06 cM for linkage groups Calb40 and Calb07.

Our sex-averaged map has high resolution, with a low average distance between adjacent markers of 0.46 cM, varying from 0.27 cM in Calb04 to 0.77 cM in Calb34. The linkage map of the close relative *C. clupeaformis*, a representative of the North American whitefish lineage, had a marker resolution across the map of 0.89 cM, around half the density of our *C. sp “Albock*” map. In the female map the average inter-marker distance was 0.48 cM varying in linkage groups (only considering linkage groups > 0 cM) from 0.31 cM in Calb04 to 0.99 cM in Calb35. The average inter-marker distance in the male map was 0.46 cM with the smallest and largest ratios found in Calb12 and Calb39 respectively with 0.18 cM and 1.05 cM.

Sex differences can be observed by comparing our sex-specific linkage maps for *C. sp. “Albock*”. Comparing total map lengths for the female and male maps gives a female:male recombination ratio of 1.09, however, this does not account for the two whitefish linkage groups which have length 0 cM in our female map (Calb38 and Calb39). Calculating this female:male recombination ratio for each linkage group separately, including only those > 0 cM in both maps, results in a ratio of 1.25. Salmonid species have been shown to have sexual dimorphisms in recombination rate with published female:male recombination ratios varying from 1.38 in Atlantic Salmon (Lien *et al.* 2011) to 2.63 in Brown Trout (Gharbi *et al.* 2006) and therefore sexual dimorphism in whitefish appears to be low in comparison to other salmonids. However, since each sex-specific linkage map represents the recombination landscape in one individual, in our case each parent of the F1 cross, more than one linkage map is required to disentangle individual variation in recombination rate and consistent sex specific recombination rate variation (Sakamoto *et al.* 2000; Moen *et al.* 2004; Lien *et al.* 2011). Although our female:male recombination ratio does not conclusively show variable recombination rates between females and males it still reveals a striking difference in map length considering the inclusion of the same set of markers for each. Studies on other teleost species, including stickleback, have also reported detailed empirical evidence of sexually dimorphic recombination rates, calculating female:male recombination ratios of linkage map lengths to be 1.64 (Sardell *et al.* 2018). Future work should aim to compare and contrast the recombination landscape of whitefish to the detailed sexually dimorphic recombination patterns observed in drosophila, mice, deer and various fish species (Dunn 1920; Sakamoto *et al.* 2000; Lenormand and Dutheil 2005; Johnston *et al.* 2017; Kubota *et al.* 2017; Sardell *et al.* 2018).

### Synteny analysis

**Figure 2:**
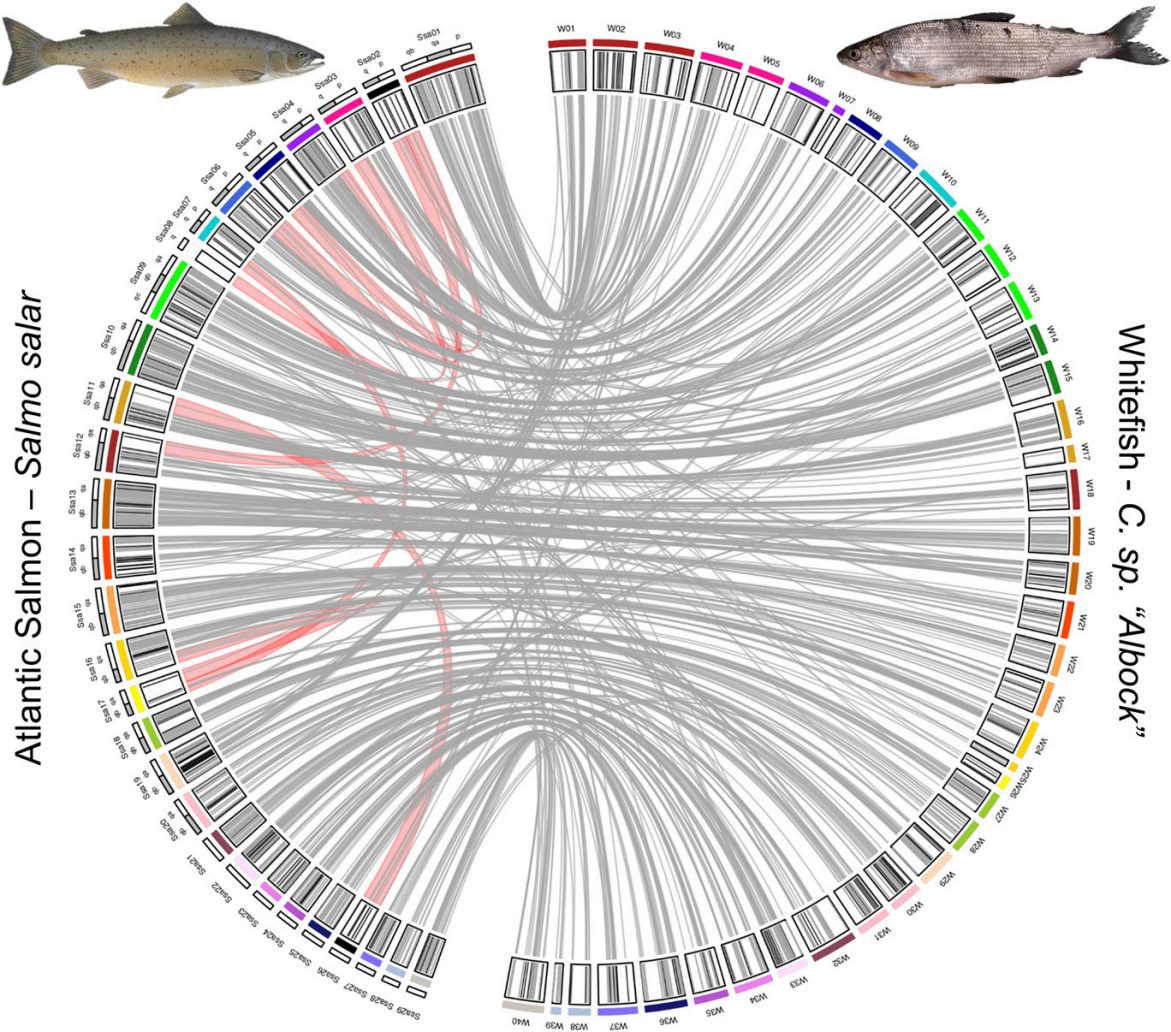
Synteny plot identifying homologous whitefish (*C. sp. “Albock*”) linkage groups and Atlantic Salmon (*Salmo salar*) chromosomes. The outermost track on the Atlantic Salmon side (left) of the plot shows the locations and names of chromosome arms (alternating in white and gray). The next track inwards shows whitefish linkage groups (right) and salmon chromosomes (left) and linkage group-chromosome synteny is denoted by the same coloring of linkage groups and chromosomes. Black salmon chromosomes Ssa02 and Ssa26 represent chromosomes with no homologous whitefish linkage groups. Salmon chromosome Ssa08 is colored in white and had no significant mappings. The innermost track highlights the location of the 839 RAD markers in the whitefish linkage map (right) which confidently map to the salmon genome (left). Those markers which map to the identified homologous chromosomes are colored in gray and those which deviate are colored in black. Links represent the mappings of 839 markers within the whitefish linkage map which were successfully mapped to the Atlantic Salmon genome. ‘Lineage-specific Ohnologue Resolution’ (LORe) regions within the salmon genome, identified by Robertson *et al.* (2017), are shown with broad red links between salmon chromosome arms.

**Figure 3:**
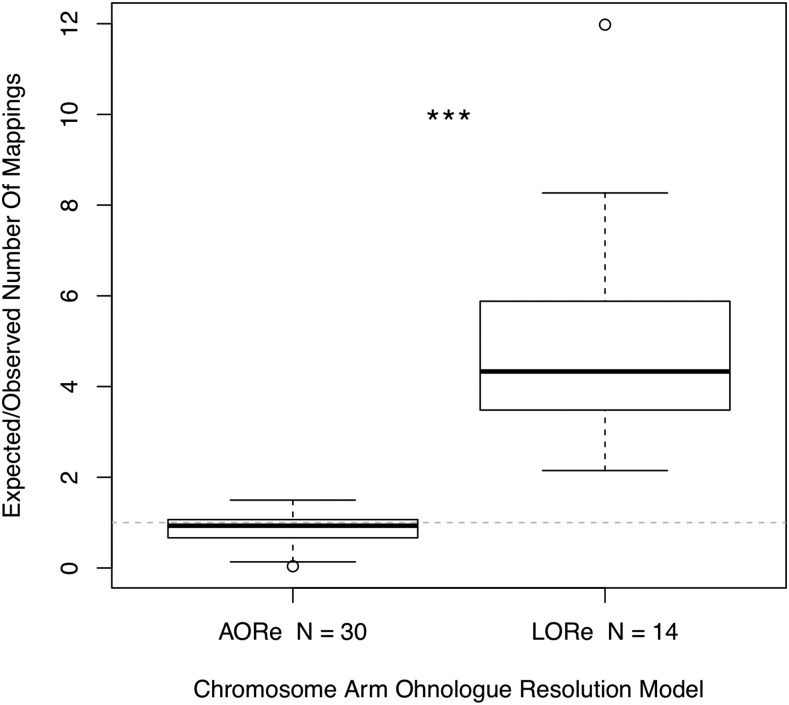
Boxplot highlighting the higher expected/observed ratio of markers mapping to the 14 ‘Lineage-specific Ohnologue Resolution’ (LORe) chromosome arms compared to the 30 ‘Ancestral Ohnologue Resolution’ (AORe) chromosome arms. The null expectation of expected mappings/observed mappings is indicated by the dotted line where expected/observed = 1. Three asterisks denote the significant difference between the expected/observed number of mappings ratio between AORe and LORe regions (Wilcoxon rank sum test: W = 0, *P* = 5.468x10^−11^).

Synteny analysis was carried out to investigate broad scale genome structural variation, such as fission and fusions of chromosomes or chromosome arms, within the Salmonidae family. Stringent filtering of mapped RAD loci to the salmon genome was applied to identify synteny while excluding uncertain mappings. From 5395 loci included in our linkage map we retained 839 mappings of high quality, which were spread across all 40 whitefish linkage groups (Figure 2). Synteny between salmon chromosomes and whitefish linkage groups was determined by identifying the most common salmon chromosome the markers on each whitefish linkage group mapped to. We also investigated the distribution of mappings along the Atlantic Salmon genome based on how rediploidization is thought to have proceeded following the Ss4R whole genome duplication at the finer chromosome arm level (Figure 3). In ‘Ancestral Ohnologue Resolution’ (AORe) regions salmon and whitefish have conserved patterns of rediploidization, which occurred in their shared ancestor resulting in a 1:1 orthology between ohnologs (Robertson *et al.* 2017). However, in ‘Lineage-specific Ohnologue Resolution’ (LORe) regions, specifically the large duplicated collinear blocks ’Ssa2p-Ssa5q’, ’Ssa2q-Ssa12qa’, ’Ssa3q-Ssa6p’, ’Ssa4p-Ssa8q’, ’Ssa7q-Ssa17qb’, ’Ssa11qa-Ssa26’ and ’Ssa16qb-Ssa17qa’ (highlighted with red links in Figure 2) identified by Robertson *et al.* (2017), rediploidization has proceeded independently in salmon and whitefish and ohnologs share a 2:2 orthology. As expected we identified that LORe regions had statistically fewer mappings than expected compared to AORe regions (Wilcoxon rank sum test: W = 0, *P* = 5.468x10^−11^) and conclude that this is the result of the mapping parameters we used (Figure 3). These parameters, aimed to identify single best mapping positions, work well in AORe regions, where we calculated that the observed number of mappings is close to the expected number (*i.e.*, a ratio of 1), meaning mappings are evenly distributed between AORe chromosome arms. Mappings to chromosome arms which make up collinear LORe blocks are not expected to be unique, lowering the mapping confidence (*i.e.*, mapping quality score) of loci there, which resulted in the filtering out of these mappings. Confident mappings within LORe regions are therefore scarce because these regions do not follow the 1:1 ohnologue orthology that we required through our mapping parameters to keep markers.

The prevalence of delayed rediploidization is likely the reason that three salmon chromosomes, Ssa02, Ssa08 and Ssa26 were not identified as homologs to any of our whitefish linkage groups, with Ssa08 having no significant mappings at all. All three of these chromosomes, specifically the Ssa08q, Ssa02p, Ssa02q and Ssa26 arms, are LORe regions and the lack of markers mapped to these regions in our analysis is likely caused by an abundance of 2:2 orthology between salmon and whitefish. Markers which might have mapped to these salmon chromosomes have likely been filtered out due to their poor mapping scores. This may also underpin the similarly uncertain assignment of synteny between the *C. clupeaformis* linkage map and these regions, carried out by Sutherland *et al.* (2016).

Only a small number of markers on each whitefish linkage group mapped to a different salmon chromosome than the identified homologous chromosome (indicated with black lines on the innermost track in Figure 2 and evidenced by the low abundance of non-parallel links from each linkage group in Figure 2). A large proportion of non-parallel links identified in our synteny analysis connect to LORe regions. However, the largest of these deviations is a series of links (16) from W02 (which was identified as homologous to Ssa01 with 18 links) to Ssa19, an AORe region. Due to the similar abundance of links to two different salmon chromosomes and the fact that rediploidization patterns in this region are shared by salmon and whitefish this series of mappings might rather reflect a whitefish specific fusion of two Atlantic Salmon chromosome arms, Ssa01qa and Ssa19.

While multiple salmonid linkage maps, including those of *C. clupeaformis* and Rainbow Trout, identified synteny from two linkage groups to one salmon chromosome for Ssa05, Ssa06, Ssa14, Ssa17 and Ssa19, we only identify synteny from one European whitefish linkage group to each of the salmon chromosomes (Ssa05-W08, Ssa06-W09, Ssa14-W21, Ssa17-W26 and Ssa19-W29; Table 1; Sutherland *et al.* 2016). Although this pattern of synteny could suggest unique genome structure in *C. sp. “Albock*” (namely that each of these linkage groups in *C. sp. “Albock*” is a fusion of two other linkage groups present in other salmonids) the patterns of synteny we observe as well as those identified by Sutherland *et al.* (2016) may be complicated by rediploidization history as indicated for multiple Atlantic Salmon chromosomes. It is now known that chromosome arms Ssa05q, Ssa06p and Ssa17qa and Ssa17qb fall within LORe regions (Robertson *et al.* 2017) and therefore the establishment of synteny relationships to these regions is challenging, especially when using a mapping approach with RAD data (90 bp only). Further work should therefore identify whether our one linkage group to one salmon chromosome pattern of synteny is consistent for W08, W09 and W26 but this would require the availability of longer sequences for synteny analysis. However, both Ssa14 and Ssa19 are within AORe regions with expected/observed ratios of mappings close to 1 and our identification of synteny from one linkage group to each of these chromosomes (W21-Ssa14 and W29-Ssa19) should not be affected by rediploidization. This pattern may therefore reflect European whitefish-specific chromosome fusions, although the mapping of some markers from W10 to Ssa14qb and similarly some markers from W02 map to Ssa19qb (as discussed above) suggests that the confident assignment of synteny between these regions will require a denser marker set.

We also identify one possible European whitefish-specific fission event with markers from both W38 and W39 mapping to Ssa28, an AORe dominated chromosome which is homologous to only one linkage group in each salmonid species compared by Sutherland *et al.* (2016) including *C. clupeaformis*. It is therefore possible that a fission event has occurred in the European whitefish lineage, however, due to relatively low number and density of markers on W38 and W39 future investigation should aim to clarify this pattern.

We identified two salmon chromosomes which were each homologous to three different whitefish linkage groups; Ssa01 to W01, W02 and W03 and Ssa09 to W11, W12 and W13 (Figure 2). These Atlantic Salmon chromosomes have been identified to map to three linkage groups in other salmonids including Brook Trout, Arctic Charr, Coho Salmon and various *Oncorhynchus* species, however, synteny with *C. clupeaformis*, the only member of Coregoninae included in these comparisons, was less clear (Kodama *et al.* 2014; Sutherland *et al.* 2016; Hale *et al.* 2017; Nugent *et al.* 2017). This syntenic pattern has been attributed to fusion events which were unique to the Atlantic Salmon lineage only. Here we add to the evidence provided by the *C. clupeaformis* linkage map that this synteny is also consistent with Coregoninae despite their significant divergence from members of the Salmoninae.

Synteny analysis between members of Salmonidae also identified a number of Atlantic Salmon chromosomes which each show synteny with two linkage groups (Sutherland *et al.* 2016; Hale *et al.* 2017). We find a similar pattern of synteny between *Salmo salar* and *Coregonus* for many of these salmon chromosomes including Ssa03 (to W04 and W05), Ssa10 (to W14 and W15), Ssa13 (to W19 and W20), Ssa15 (to W22 and W23), Ssa16 (to W24 and W25), Ssa18 (to W27 and W28) and Ssa20 (to W30 and W31) (Figure 2). In addition to these, our synteny analysis also identified Ssa04 as homologous to W06 and W07 and Ssa11 as homologous to W16 and W17. However, links from W07 and W17 map to the LORe regions Ssa04p and Ssa17qa, and Ssa11qa and as with other salmon chromosomes within LORe regions this complicates the assignment of synteny. Although we can be confident that W06 is homologous to Ssa04q and W16 to Ssa11qb, since both of these chromosome arms are AORe regions, the dominance of LORe in Ssa04p and Ssa11qa complicates the assignment of synteny with W07 and W17. We also find that the multiple one to one relationships between salmon chromosomes and salmonid linkage groups identified by Sutherland *et al.* (2016) are also consistent with our map including those to Ssa12 (W18), Ssa22 (W33), Ssa23 (W34), Ssa24 (W35), Ssa25 (W36), Ssa27 (W37) and Ssa29 (W40; Table 1).

Two salmon chromosomes, Ssa07 and Ssa21 were shown by Sutherland *et al.* (2016) to have synteny to two linkage groups in *C. clupeaformis* but only one linkage group in all other salmonids. Our *C. sp. “Albock*” map identifies synteny from only one linkage group, W10, to Ssa07 and similarly from W32 to Ssa21 suggesting the pattern of synteny may not be conserved between *Coregonus* species. Since Ssa07q is a LORe dominated chromosome arm the lack of synteny identified to a second whitefish linkage group may be the result of the lack of 1:1 ohnolog orthology and therefore a lack of confident mappings. The pattern of Ssa21 on the other hand most likely represents a difference between *C. cluepeaformis* and *C. sp. “Albock*” since Ssa21 has an expected/observed mappings ratio of 0.94 (close to 1) and a high density of markers. Further work must therefore be carried out to better identify potential genome structural variation between *C. sp. “Albock*” and *C. clupeaformis*.

Both broad and small scale structural variations, including inversions, duplications and deletions, have been observed between closely related species and the mis-segregation which can occur during meiosis as a result of these variations is thought to be able to play a role in the speciation process (Feulner and De-Kayne 2017). It is therefore possible that European and North American whitefish lineages (and even species within these lineages) have unique structural variations which may underpin reproductive isolation in sympatry. Without more detailed information on genome wide synteny and the occurrence of structural variation between these two lineages it is difficult to determine whether the observed variation in synteny patterns to the Atlantic Salmon (*e.g.*, with regards to Ssa14, Ssa19, Ssa21 and Ssa28) represents true variation between these species or variation in linkage mapping resolution and accuracy. A comparison of synteny between our *C. sp. “Albock*” map and the Atlantic Salmon (using our synteny mapping approach) and the *C. clupeaformis* map to the Atlantic Salmon (compared by Sutherland *et al.* 2016) can be found in Table S1.

### The development of genomic resources for European whitefish

A wealth of genomic resources used to study adaptation and speciation are now available for a variety of systems. Multiple species from popular model radiations including Galapagos finches (Lamichhaney *et al.* 2015) and Lake Victoria cichlids (Brawand *et al.* 2014) now have highly contiguous, well curated and annotated, reference genomes. These resources provide the opportunity to ask specific questions about intra and inter-species genomic differences with many studies focusing on understanding the genomic basis of adaptation and reproductive isolation. Studies can now utilize high throughput whole-genome sequencing to achieve high depth of coverage and are able to map these reads to a reference genome to understand the distribution of genomic variation along the genome. However, many interesting organisms including the many ecologically diverse salmonids have only a handful of highly contiguous and well annotated reference genomes available. Current well annotated salmonid genomes include those of Atlantic Salmon (*Salmo salar*; Lien *et al.* 2016) and Rainbow Trout (*Oncorhynchus mykiss*; Berthelot *et al.* 2014). However, recently assemblies of Chinook Salmon (*Oncorhynchus tshawytscha*; Christensen *et al.* 2018), Coho Salmon (*Oncorhynchus kisutch*; NCBI BioProject: PRJNA352719), Arctic Charr (*Salvelinus alpinus*; NCBI BioProject: PRJNA348349; Christensen *et al*. 2018) and Grayling (*Thymallus thymallus*; Varadharajan *et al.* 2018) have also been published. Although these genomes expand the diversity of salmonid genomes available dramatically, they are still relatively distantly related to the diverse whitefish subfamily Coregoninae.

Our linkage map fills a gap in the resources available to analyze European whitefish genetic data allowing investigation into this species rich, ecologically diverse, lineage. The patterns of synteny between European whitefish and Atlantic Salmon reported here should be further investigated once whitefish genomes become available to identify synteny at a finer scale, identifying chromosome fission and fusion events and possible inversions also within the *Coregonus* genus. Our linkage map can also be paired with future resources to investigate the outcome of whole genome duplication including estimations of the rediploidized proportion of the genome, already calculated in Atlantic Salmon. Future work should further aim to identify regions of the genome which may underpin reproductive isolation in whitefish to better understand the speciation mechanism in this adaptive radiation.

In conclusion, we have produced the densest *Coregonus* linkage map to date, with a total sex-averaged map length of 2293.86 cM containing 5395 SNP loci. We have found evidence of sex-specific recombination rate variation within *C. sp. “Albock*” by calculating the female:male recombination ratio *i.e.*, a ratio of female and male linkage map lengths. The level of heterochiasmy inferred by this ratio is reflected in other species with known sex-specific recombination variation, including other salmonids (Gharbi *et al.* 2006; Lien *et al.* 2011). We also show that *C. sp. “Albock*” linkage groups exhibit synteny with Atlantic Salmon chromosomes, in some cases following a pattern of synteny shared with other salmonid species. This linkage map will facilitate a host of future studies into the genomic basis of adaptation in Alpine whitefish including those on the identification of QTL for traits of interest, the interpretation of genome-wide divergence data and the colocalization of regions under selection *e.g.*, F_ST_ outliers identified from genome scans. It also has the potential to assist in the ongoing assembly of Alpine whitefish reference genomes.
